# Lipoxin A4 attenuated dexamethasone-induced muscle atrophy via activation of PGC-1α/Nrf2/TFAM pathway

**DOI:** 10.1007/s13105-022-00925-1

**Published:** 2022-09-20

**Authors:** Fatma H. Rizk, Nema A. Soliman, Shaimaa M. Kashef, Amira A. Elsaadany

**Affiliations:** 1 Department of Physiology, Faculty of Medicine, Tanta University, Tanta, Egypt; 2 Department of Medical Biochemistry, Faculty of Medicine, Tanta University, Tanta, Egypt; 3 Department of Histology and Cell Biology, Faculty of Medicine, Tanta University, Tanta, Egypt; 4 Department of Pharmacology, Faculty of Medicine, Tanta University, Tanta, Egypt

**Keywords:** Dexamethasone, Skeletal muscle atrophy, Lipoxin A4, Mitochondrial function, Oxidative stress

## Abstract

Prolonged dexamethasone (DEX) administration causes skeletal muscle atrophy through induction of both oxidative stress and mitochondrial dysfunction. Lipoxin A4 (LXA4) is a recognized antioxidant but its effect against DEX-induced muscle atrophy has not been studied yet. This study aimed to assess the potential ameliorating effect of LXA4 on DEX-induced muscle atrophy and investigate the possible involvement of the mitochondrial dynamics pathway and the redox state in this effect. Forty male rats were divided into four groups; normal control, LXA4-treated, DEX-treated, and LXA4 plus DEX-treated. At the end of the experiment, LXA4 counteracted the effect of DEX on different parameters including muscle weight, muscle strength, serum creatine kinase activity, malondialdehyde and protein carbonyl contents, Na/K-ATPase and citrate synthase activities, mitochondrial transmembrane potential, mitochondrial transcription factor (TFAM), peroxisome proliferator-activated receptor gamma coactivator 1-alpha (PGC-1α), and nuclear factor erythroid 2-related factor 2 (Nrf2). These findings signify the promising therapeutic effect of LXA4 against DEX-induced skeletal muscle atrophy and indicate the possible involvement of LXA4-induced mitochondrial activation in addition to its well-known antioxidant effects.

## Introduction

Skeletal muscle atrophy is a pathological condition characterized by loss of muscle mass and decreased muscle strength leading to weakness, fatigue, and impairment of the quality of life [13]. Many diseases are associated with muscle atrophy including diabetes mellitus, sepsis, chronic kidney disease, disuse conditions, and metabolic acidosis [26].

Moreover, high doses and/or prolonged administration of dexamethasone (DEX) induces muscle atrophy and increases morbidity and mortality, which may limit its clinical use [28]. Mitochondrial dysfunction [18] and oxidative stress have been proven to mediate DEX-induced muscle atrophy [2]. Thus, drugs that improve mitochondrial function and redox state might ameliorate DEX-induced muscle atrophy.

Several compounds have been studied for their protective effects against DEX-induced muscle atrophy, such as growth factors, androgens, taurine, glutamine, and beta-adrenergic agonists [26], but none of them has shown complete safety and efficacy. Hence, there is a pressing need to develop safe, effective agents against DEX-induced muscle atrophy.

Lipoxin A4 (LXA4) is an endogenous lipid mediator synthesized by the action of lipoxygenase enzyme on arachidonic acid. It has been found to improve mitochondrial dysfunction and reduce oxidative stress in gastric ulcer models [21]. However, no reports are available regarding its effect on skeletal muscle atrophy.

Normal mitochondrial function depends mainly on mitochondrial biogenesis and dynamics. Peroxisome proliferator-activated receptor γ coactivator 1α (PGC-1α) is the critical regulator of mitochondrial biogenesis. It modulates mitochondrial dynamics via the regulation of gene expression of mitochondrial DNA that encodes intramitochondrial proteins [22]. Dexamethasone suppresses PGC-1α expression levels. Accordingly, treatments that increase PGC-1α levels in muscles shall preserve the normal mitochondrial function and prevent DEX-induced muscle atrophy [10]. Therefore, the current study aimed to assess the potential mitigating effect of LXA4 on DEX-induced muscle atrophy and investigate the possible involvement of the mitochondrial dynamics pathway and the redox state in this effect.

## Material and methods

### Drugs and chemicals

Dexamethasone (ampules) was obtained from the Amriya Pharmaceutical Industries, Egypt. Lipoxin A4 (89,663–86-5), as well as the components of the phosphate buffer saline and mitochondrial isolation buffer, was purchased from Sigma-Aldrich Co, St. Louis, MO, USA.

### Animals

Forty male Wistar rats weighing 170–200 g were obtained from the official animal supplier of the Faculty of Medicine, Tanta University. The study was performed at the Pharmacology Department, Faculty of Medicine, Tanta University in January 2021. The animals were kept in well-ventilated cages (5 rats per cage) under strict hygienic measures, at 25 ± 2 °C room temperature with 12/12 h light/dark cycles, and they were allowed free access to commercial rodent chow (EL-Nasr Chemical Company, Cairo, Egypt) and tap water. The rats were acclimatized for 2 weeks before the start of the experiment. All procedures were performed during the same time of the day. The rats were randomly divided into four groups (10 animals each) and were treated once daily for 14 days. Group I (normal control group) received saline subcutaneously in the back of the rat and intraperitoneally in the same doses that the drugs were given to groups II and III [9]. Group II (LXA4-treated group) received LXA4 (1 μg/kg) via intraperitoneal injections [11]. Group III (DEX-treated group) received DEX (2 mg/kg) by subcutaneous injections in the back of the rats [9]. Group IV (LXA4 + DEX-treated group) concomitantly received LXA4 and DEX in the same doses and via the same routes as groups II and III, respectively [9, 11].

### Assessment of muscle strength using four limb hanging test on the 13^th ^day of the experiment

The four limb hanging test assessed the ability of the rat to produce sustained tension in the limb muscles. The rat was allowed to grasp aluminum mesh with its four paws; then, the mesh was inverted, and the hang period started with all four paws of the rat grasping the mesh and stopped by falling off the animal or reaching a maximum of 120 s holding the wire. Three trials were allowed for each animal with 5 min inter-trial intervals [29]. The results were represented as (A) the hang time (in seconds) that represented the mean hanging time of the three trials, calculated by adding the hang periods of the three trials divided by three; and (B) the holding impulse, which reflected the tension (impulse) that the animal developed for maintaining itself on the wire against the effect of the gravity for the longest period, calculated using the following formula [9]: 

$$\mathrm{holding}\;\mathrm{impulse}\;(\mathrm s\ast\mathrm g)\:=\:\mathrm{hang}\;\mathrm{time}\;(\mathrm s)\:\times\:\mathrm{body}\;\mathrm{weight}\;(\mathrm g)$$

At the end of the experiment, the overnight fasting rat was weighed to calculate the percentage of change in its body weight. The rat was then euthanized by diethyl ether, and its blood was collected in sterile tubes by cardiac puncture and centrifuged for 10 min at 3000 rpm. Serum creatine kinase (CK) activity was assayed using the MAK116 assay kit (Sigma-Aldrich Co, St. Louis, MO, USA). Gastrocnemius muscles were quickly collected for further analyses.

### Tissue collection and preparation

Gastrocnemius muscles were dissected, weighed, and cut into pieces. One piece was immersed in neutral buffered formalin 10%, fixed, and stained with hematoxylin and eosin to be examined for morphological changes. The second piece was used for nuclear extraction using the Nuclear/Cytosol Fractionation Kit (266‐25) (BioVision, Inc, CA, USA). The third piece was homogenized in cold phosphate-buffered saline (pH 7.4), then centrifuged at 3000 rpm for 20 min for the assay of cytosolic biomarkers, and the fourth piece was immediately washed with phosphate-buffered saline and homogenized in 1 ml of mitochondrial isolation buffer (0.01 mol/liter Tris–HCl, 0.0001 mol/liter EDTA-2Na, 0.01 mol/liter sucrose, 0.8% NaCl, pH 7.4) using a glass grinding tube on ice for 20 times. The resultant homogenate was centrifuged at 1500 rpm for 10 min at 4 °C, and the supernatant was collected and centrifuged again at 10,000 rpm for 15 min. The precipitate obtained represented the mitochondria fraction of gastrocnemius muscle according to the method described by Ding et al. [5]. The resultant nuclear and cytosolic extracts were frozen at − 80 °C until being used for biochemical analysis. The residual pieces were stored at − 80 °C to be used for molecular and Na/K-ATPase activity analysis. The tissue protein content was determined by the method of Lowery et al. [20].

### Assessment of oxidant status biomarkers

Oxidative damage of lipids and proteins was quantified through colorimetric assays of malondialdehyde (MDA) and protein carbonyl (PCC) contents in muscle tissue homogenates. Malondialdehyde was assayed using a commercial kit supplied by Biodiagnostic, Giza, Egypt [23], whereas PCC was measured as described previously [17]. Both levels were expressed as nmol/mg protein. The colorimetric assays were done using the Biosystem spectrophotometer (BTS 350 semiautomatic analyzer, Mart and Medical Services, New Delhi, India).

### Na/K-ATPase activity assay

The Na/K-ATPase activity was assayed using a commercial kit (MBS8243226) supplied by MyBiosource, Inc., San Diego, USA. Briefly, 0.1 g from gastrocnemius muscle was homogenized with 1 ml of the assay buffer on ice and centrifuged at 8000 rpm for 10 min at 4 °C. The supernatant was taken into a new centrifuge tube and kept on ice for detection of Na/K-ATPase activity on a plate reader at a wavelength of 660 nm [8].

### Citrate synthase (CS) activity assay

In a freshly prepared mitochondrial extract, CS activity was measured using 5,5′‐dithiobis (2‐nitrobenzoic acid) using a spectrophotometer (absorbance at 412 nm) overtime at 30 °C in the presence of acetyl co‐enzyme A and oxaloacetate [15]. The enzyme activity was expressed as nmol/min/mg protein.

### Measurement of mitochondrial transmembrane potential (ΔΨm)

The ΔΨm was measured as described by Madi et al. [21] and Guha et al. [7] using the cationic carbocyanine dye (JC-1) (HY-15534) (MedChemExpress, New Jersey, USA). In brief, freshly prepared mitochondria (20 µg) isolated from gastrocnemius muscle were incubated with JC-1 for 10 min in the dark at 37 °C. The JC-1 dye exhibited a potential-dependent accumulation in the mitochondria indicated by a fluorescence emission shift from green at 529 nm wavelength to red at 590 nm wavelength. Accordingly, the ΔΨm was designated by a decrease in the red/green fluorescence intensity ratio. The potential-sensitive color shift was due to the concentration-dependent formation of red fluorescent J-aggregates (different aggregate formation rates).

### Immunoassay

An enzyme-linked immunosorbent assay was used to estimate mitochondrial transcription factor A (TFAM) (MBS942857) and PGC-1α (MBS762203) levels in muscle nuclear extracts using commercial kits according to the manufacturers’ instructions (MyBiosource, Inc., San Diego, USA). Color development was monitored by absorbance at 450 nm using a microplate reader (Stat Fax 2100, New York, USA).

### Relative gene expression of nuclear factor erythroid 2-related factor 2 (Nrf2)

The frozen muscle tissues were processed, and the total RNA was extracted using the Qiagen RNeasy Total RNA isolation kit (74,104) according to the manufacturer’s guidelines (Qiagen, Hiden, Germany). This was followed by the synthesis of the first strand using the SuperScript III First‐Strand Synthesis System for RT‐PCR kit (18,091,050) (Thermo Fisher Scientific, USA). The PCR reactions were performed using Power SYBR Green PCR Master Mix (4,368,577) (Thermo Fisher Scientific, USA). The *Nrf2* mRNA expression was measured in relation to the housekeeping gene, *Glyceraldehyde 3-phosphate dehydrogenase (GAPDH)*. The primers were designed using Primer3 software (http://bioinfo.ut.ee/primer3/) as follows: rat Nrf2 forward primer (5′-TAGCAGAGCCCAGTGGCGGT-3′) and reverse primer (5′-TGCTCTGGGGATGCTCGGCT-3′) (GenBank Accession No. NM_031789.2); rat GAPDH forward primer (5′‐GGTGAAGTTCGGAGTCAACGGA‐3′) and reverse primer (5′‐GAGGGATCTCGCTCCTGGAAGA‐3′) (GenBank Accession No. NM_017008) [1]. The relative gene expression was automatically calculated using the comparative threshold (Ct) method [19] for the values of the target and the reference genes by Rotor‐Gene Q 6plex and its specific software (Qiagen, Valencia, CA, USA).

### Histopathological study

Specimens from gastrocnemius muscles were fixed in 10% formal saline for 24 h, dehydrated in ascending series of ethyl alcohol, and embedded in paraffin. Five μm histological sections were cut, stained with hematoxylin and eosin [6], and examined using a Leica light microscope with a built-in camera.

### Measurement of cross-sectional area (CSA)

Over 200 myofibers per one gastrocnemius muscle were measured manually, and the mean of the cross-sectional area (CSA) was utilized using ImageJ software (National Institute of Health, Bethesda, MD, USA) in a blinded manner to calculate the average CSA for each group [9].

### Statistical analysis

Statistical tests were performed using the SPSS software program (IBM SPSS Statistics for Windows, IBM Corp, version 23.0. Armonk, NY, USA). The data were expressed as mean ± standard deviation (SD). Statistical comparisons between different groups were conducted using the one-way analysis of variance (ANOVA) test followed by LSD post hoc test for multiple comparisons. *P* values < 0.05 were considered statistically significant.

## Results

### Effect of LXA4 and DEX on body weight

**Fig. 1:**
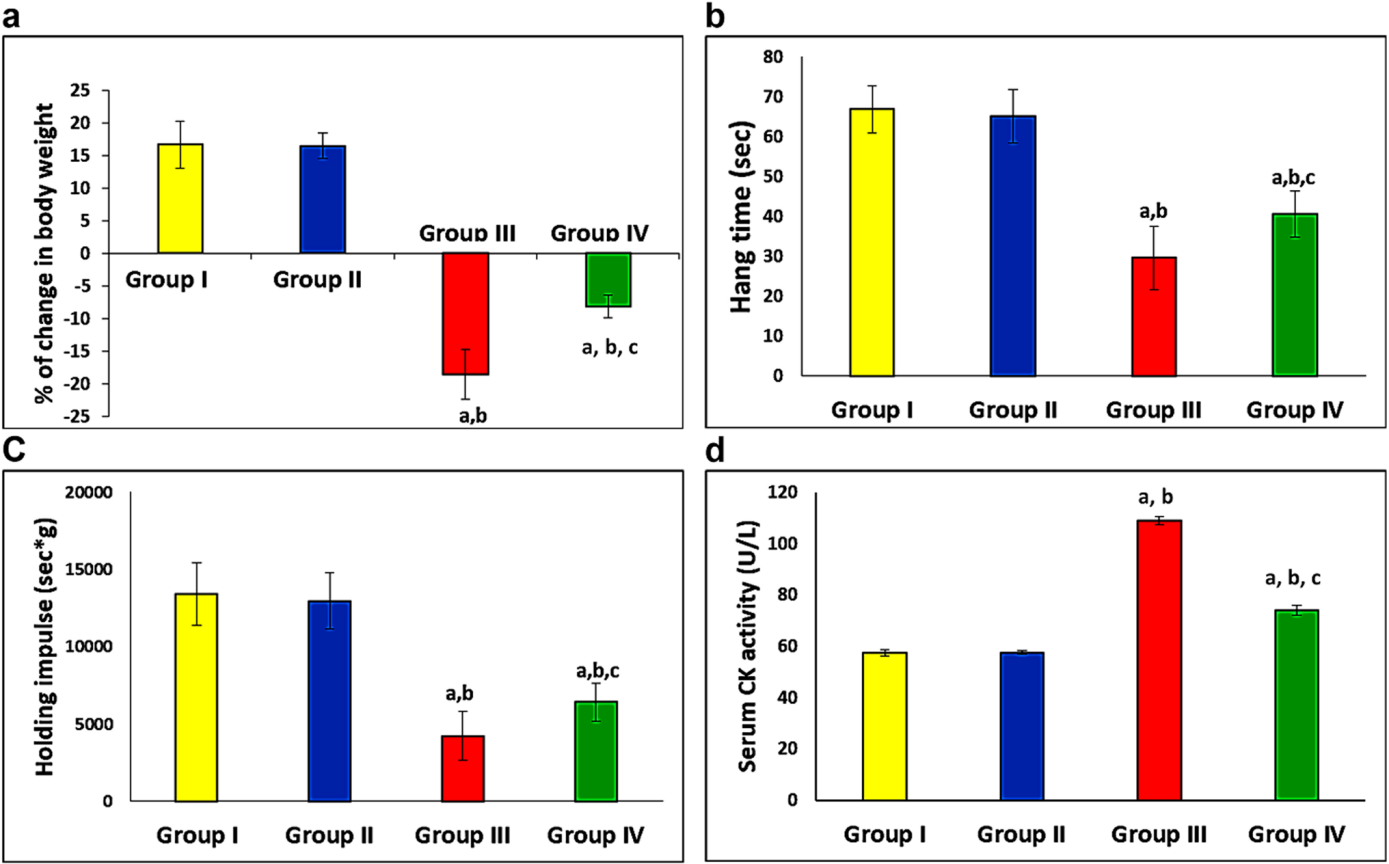
LXA4 attenuated DEX-induced muscle atrophy. **a** % of change in body weight. It equals the difference between rat weight at the start and end of the experiment/rat weight at end of experiment × 100. **b**, **c** demonstrate muscle strength by calculating the mean hang time and holding impulse. **d** Serum creatine kinase (CK) activity. Group I, normal control group; group II, LXA4-treated group; group III, DEX-treated group; and group IV; LXA4 + DEX-treated group; *n* = 10 for each group. Data are expressed as mean ± SD, ^a^*p* < 0.05 vs group I, ^b^*p* < 0.05 vs group II, and ^c^*p* < 0.05 vs group III

By the end of the experiment, the body weight of the control and LXA4 groups increased by 16.66% and 16.49%, respectively, compared to their initial body weight. Compared to the control group, DEX administration significantly decreased the body weight by about 18.59% of the initial body weight. Concomitant administration of LXA4 with DEX significantly attenuated the decrease in body weight in comparison with the DEX group as it decreased by about 8.14% of the initial body weight. These findings suggested that LXA4 treatment significantly decreased muscle atrophy (Fig. 1a).

### Effect of LXA4 and DEX on muscle strength

Administration of DEX for 14 days significantly decreased the hang time and holding impulse as compared with the normal control group, while concomitant administration of LXA4 with DEX significantly increased them as compared with the DEX group. Administration of LXA4 for 14 days did not affect the hang time or the holding impulse significantly as compared with the control group. These findings indicated that LXA4 treatment may preserve normal muscle function (Fig. 1b, c).

### Effect of LXA4 and DEX on serum CK activity

Serum CK activity significantly increased in the DEX group as compared with the normal control group. Concomitant administration of LXA4 with DEX significantly decreased it as compared with the DEX group, which indicated a reduction of muscle damage by LXA4 treatment. LXA4 administration alone did not affect serum CK activity significantly (Fig. 1d).

### Effect of LXA4 and DEX on oxidant status biomarkers

Dexamethasone administration significantly increased both MDA and PCC levels in gastrocnemius muscles as compared with the normal control group. Concomitant administration of LXA4 with DEX significantly decreased them as compared with the DEX group. Administration of LXA4 did not lead to any significant changes in MDA or PCC levels as compared with the control group (Table 1).

**Table 1:** Effect of LXA4 and DEX on oxidant status biomarkers in gastrocnemius muscles

	Group I (*n* = 10)	Group II (*n* = 10)	Group III (*n* = 10)	Group IV (*n* = 10)
MDA (nmol/mg protein)	1.28 ± 0.14	1.25 ± 0.11	3.66 ± 0.32^a,b^	2.20 ± 0.15^a,b,c^
PCC (nmol/mg protein)	2.45 ± 0.18	2.43 ± 0.26	5.20 ± 0.18^a,b^	3.23 ± 0.30^a,b,c^

LXA4 attenuates DEX-induced oxidative stress in gastrocnemius muscles. Group I, normal control group; group II, LXA4-treated group; group III, DEX-treated group; and group IV, LXA4 + DEX-treated group. *MDA* malondialdehyde, *PCC* protein carbonyl content, *n* number of rats in each group. Data are expressed as mean ± SD. ^a^*p* < 0.05 vs group I, ^b^*p* < 0.05 vs group II, and ^c^*p* < 0.05 vs group III

### Effect of LXA4 and DEX on Na/K-ATPase activity in gastrocnemius muscles

**Fig. 2:**
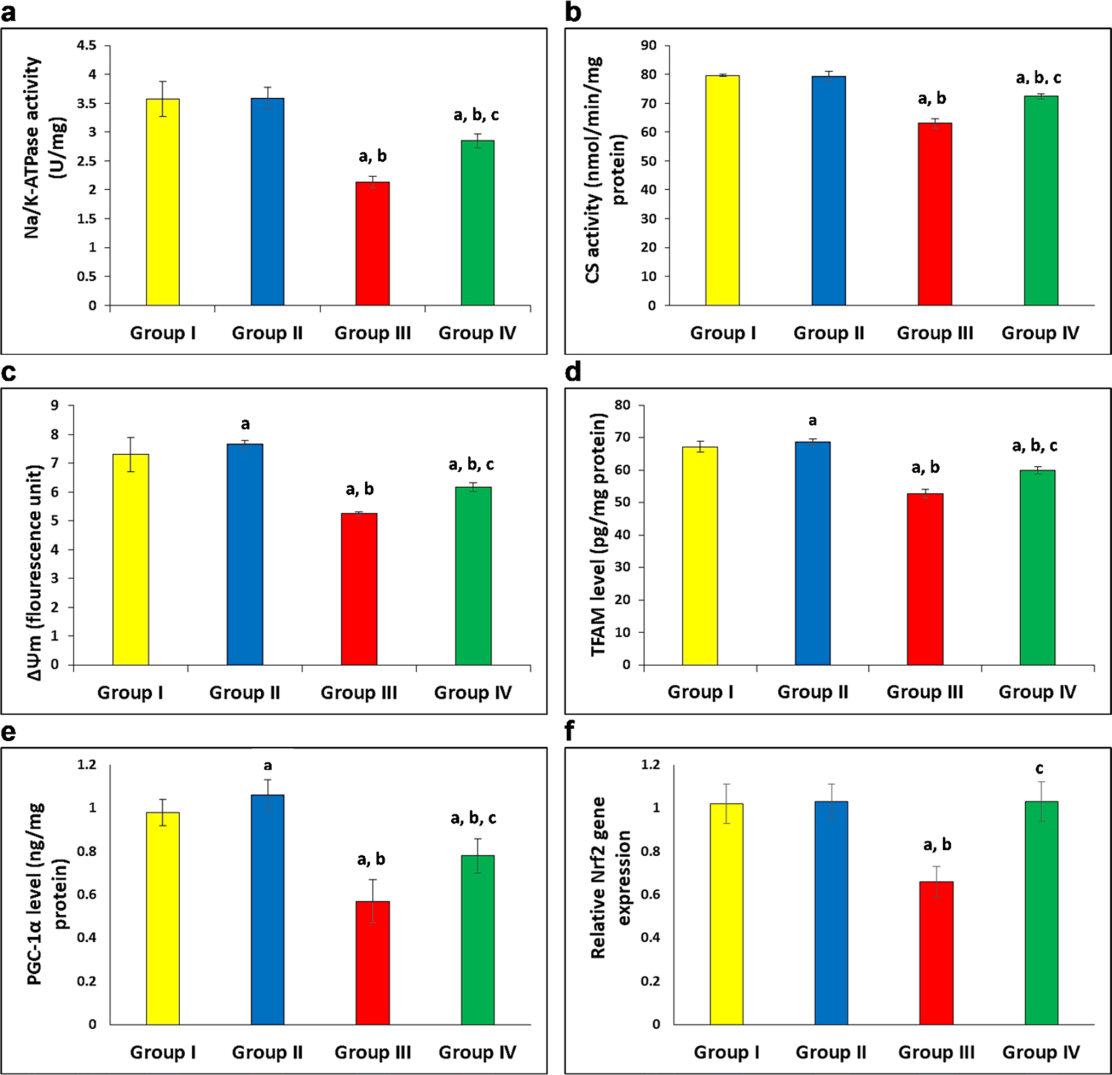
LXA4 improved Na/K-ATPase activity and mitochondrial functions in gastrocnemius muscles. **a** Na/K-ATPase activity. **b** Citrate synthase (CS) activity. **c** Mitochondrial transmembrane potential (ΔΨm) in fluorescence unit. **d** Mitochondrial transcription factor (TFAM). **e** Peroxisome proliferator-activated receptor gamma coactivator 1-alpha (PGC-1α). **f** Nuclear factor erythroid 2-related factor 2 (Nrf2). Group I, normal control group; group II, LXA4-treated group; group III, DEX-treated group; and group IV, LXA4 + DEX-treated group; *n* = 10 for each group. Data are expressed as mean ± SD, ^a^*p* < 0.05 vs group I, ^b^*p* < 0.05 vs group II, and ^c^*p* < 0.05 vs group III

There were significant decreases in Na/K-ATPase activity in gastrocnemius muscles of the DEX group as compared with the normal control group, while the combination of LXA4 with DEX significantly increased its activity as compared with the DEX group (Fig. 2a).

### Effect of LXA4 and DEX on mitochondrial functions in gastrocnemius muscles

There was impairment of mitochondrial functions as indicated by significant decreases in CS activity, ΔΨm, TFAM level, PGC-1α level, and relative gene expression of *Nrf2* in gastrocnemius muscles of the DEX group as compared with the control group. The combination of LXA4 with DEX significantly increased them as compared with the DEX group and relative gene expression of *Nrf2* reached a normal level. In addition, LXA4 significantly increased ΔΨm, TFAM level, and PGC-1α levels in gastrocnemius muscles of group II as compared with group I (Fig. 2b, c, d, e, f).

### Histopathological analysis of the effect of LXA4 and DEX on the structure of gastrocnemius muscles

**Fig. 3:**
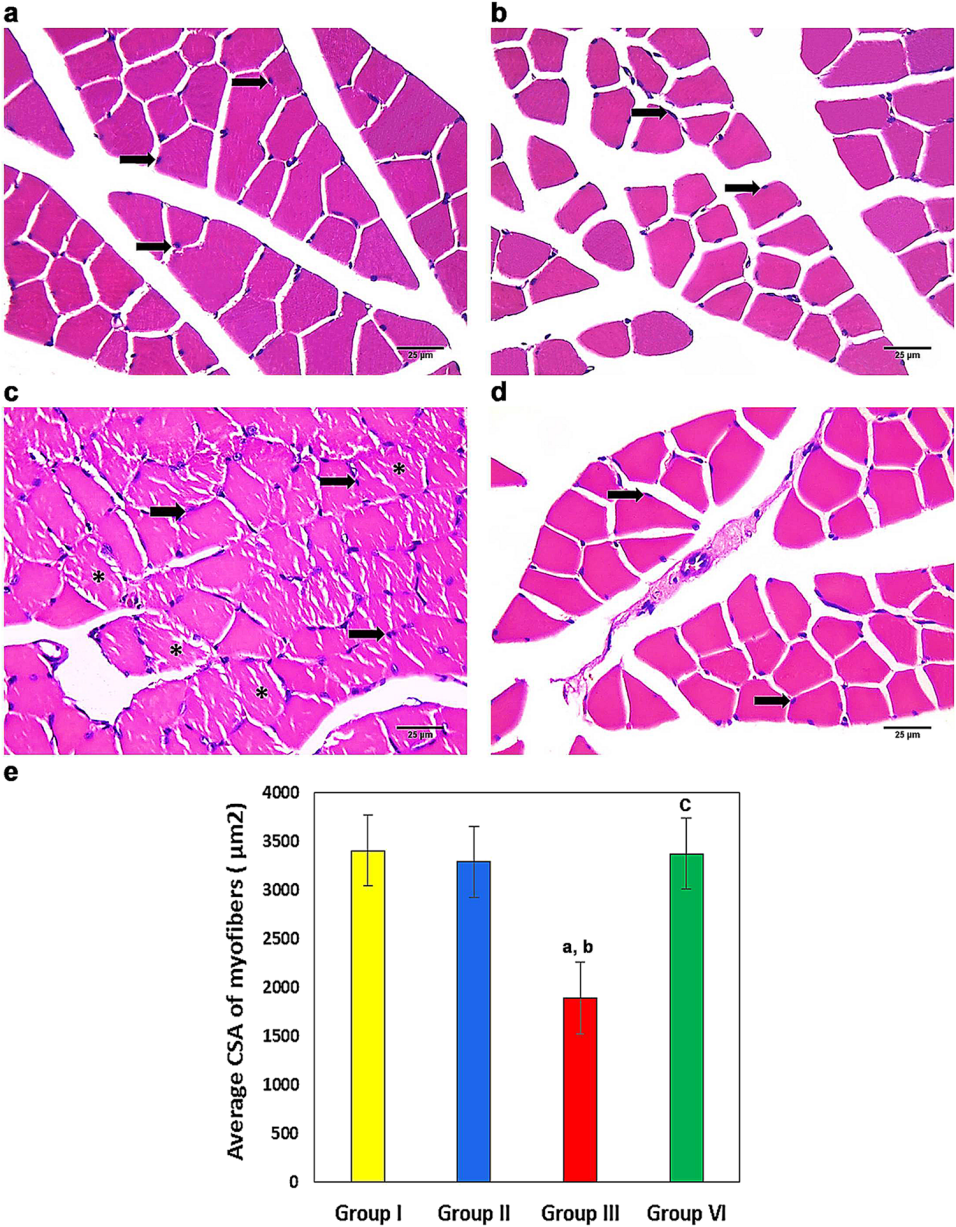
H&E staining: **a**, **b** groups I and II (normal control and LXA4) showing the polygonal fibers with peripheral nuclei (arrow). **c** Group III (DEX group) showing shrunked fibers (*) with reduced diameter and increased nuclei (arrow). **d** Group IV (DEX + LXA4) showing restoration of normal polygonal appearance of muscle fibers with peripheral nuclei (arrow). (H&E × 400, scale bar = 25 μm). **e** Histogram of the mean cross-sectional area (CSA) of myofibers in μm^2^. LXA4 prevents DEX-induced muscle atrophy and increases the mean CSA of myofibers. Data are expressed as mean ± SD, *n* = 10, ^a^*p* < 0.05 vs group I, ^b^*p* < 0.05 vs group II, and ^c^*p* < 0.05 vs group III

Microscopic examination of hematoxylin and eosin-stained sections from gastrocnemius muscles showed the normal structure of muscle fibers in the form of polygonal fibers with peripheral nuclei in groups I and II (Fig. 3a, b). Muscle sections from DEX-treated animals exhibited atrophic changes with a reduction in the fiber diameters and an increase in the nuclei numbers. Many shrunken, irregular-shaped fibers were seen with loss of their characteristic polygonal appearance (Fig. 3c). On the contrary, most of the muscle fibers from group IV (DEX + LXA4) showed restoration of the normal structure (Fig. 3d). The morphometric study confirmed these histological findings, where the mean CSA of myofibers was significantly decreased in the DEX group as compared with groups I and II. Moreover, group VI (DEX + LXA4) showed a significant increase in the mean CSA of myofibers as compared with the DEX group (Fig. 3e).

## Discussion

The present study exhibited that LXA4 administration for 14 days significantly mitigated the DEX-induced muscle atrophy in rats as shown by biochemical and histological findings. Noteworthy, this is the first study to investigate the protective effect of LXA4 against DEX-induced muscle atrophy in rats and elucidate new mechanisms involved in the effect of LXA4 on mitochondrial dynamics that has not been well identified yet.

The results of the present experiment showed that DEX-treated rats had a significantly increased percentage of loss of body weight and decreased muscle strength as indicated by the shortening of the hang time and reduction of the holding impulse compared with the normal control rats. These findings agree with the results of Huang et al. [10] who reported that DEX significantly decreased the total body weight, the gastrocnemius and tibialis anterior muscles weights, and the muscle/body weight ratio as well as the muscle strength. The reduction of body weight and muscle strength in this group could be explained by the DEX-induced catabolic action [24], where it activates catabolic (e.g., ubiquitin-proteosome and lysosomal) and suppresses anabolic (e.g., insulin-like growth factor-1/AKT/mammalian target of rapamycin [IGF-1/AKT/mTOR]) pathways that control muscle protein metabolism [26]. In the current study, the catabolic effect of DEX was indicated by the increased serum CK activity, which is a marker of muscle damage. In addition, histological examination in the DEX-treated animals revealed a loss of the myofibers’ characteristic polygonal appearance, an increase in its nuclei number, and reduction of its CSA compared with the normal control group. The increased number of myofibers’ nuclei that occurred with muscle damage may be due to activation of the satellite cells on the periphery of the muscle fibers, followed by proliferation, differentiation, and fusion into the damaged myofibers. This fusion adds nuclei to the existing fibers to compensate for the damaged nuclei [3].

Concomitant administration of LXA4 and DEX significantly reduced the muscles’ atrophic changes and improved their strength as indicated by the reduction of body weight loss and serum CK activity as well as the increase of myofibers’ CSA, hang time, and holding impulse compared with DEX group.

The possible mechanisms of the LXA4-induced protective effect may be suggested in light of the results of this study. Administration of LXA4 reduced the elevated muscles’ MDA and PCC levels, indicating that the DEX-induced oxidative damage of the muscles’ lipids and proteins was significantly attenuated by LXA4.

Dexamethasone has been reported to induce oxidative stress and mitochondrial dysfunction [10]. Mitochondria are the main cellular source of ATP production, reactive oxygen species (ROS) generation, and scavenging [4]. The excessive production of mitochondrial ROS induces oxidative stress and causes damage to DNA, lipids, and proteins of the mitochondria and other cellular organelles with subsequent exacerbation of mitochondrial dysfunction and further damage to the cells and tissues [12]. In the present study, the decrease in Na/K-ATPase activity in the DEX group compared with the control group could be attributed to oxidative stress and energy deprivation caused by disturbances in mitochondrial functions, which in turn decreased the enzyme activity [22].

Dexamethasone-induced mitochondrial dysfunction was demonstrated in this study by the decrease in CS activity and ΔΨm compared with the normal control group. This could be explained by the overproduction of mitochondrial ROS, which impaired enzyme functions in the respiratory chain and reduced mitochondrial biogenesis [27].

On the other hand, LXA4 improved mitochondrial functions as indicated by the significant increase in Na/K-ATPase activity, CS activity, and ΔΨm compared with the DEX group. This improvement may be due to the activation of the PGC-1α/Nrf2/TFAM pathway, which was inhibited by DEX administration. PGC-1α is a master regulator of mitochondrial biogenesis, which activates many transcription factors that bind to the promoters of nuclear-encoded mitochondrial genes [25]. It’s well known that PGC-1α coactivation of Nrf2 promotes the expression of TFAM, which stimulates mitochondrial DNA synthesis and gene transcription [16]. Furthermore, PGC-1α regulates oxidant-antioxidant homeostasis of the cells by upregulating gene expression of the superoxide dismutase-2, glutathione peroxidase1, catalase, and uncoupling proteins. So, PGC-1α played an important role in reducing ROS-induced damage by stimulating the activity of antioxidant enzymes and increasing the uncoupling capacity, which in turn reduced mitochondrial ROS production [14].

## Conclusion

It could be concluded that LXA4 displayed a protective effect against DEX-induced muscle atrophy in rats, possibly through the improvement of mitochondrial functions and activation of the PGC-1α/ Nrf2/TFAM pathway in addition to its antioxidant activity. So, LXA4 may be considered a promising therapeutic agent for DEX-induced muscle atrophy. Future studies are needed to investigate the therapeutic effects of LXA4 regarding other types of muscle atrophy and assess the efficacy of concomitant administration of LXA4 and DEX in patients on long-term corticosteroid therapy.

## Data Availability

All data are included in this article.
